# Sepsis-induced AKI: From pathogenesis to therapeutic approaches

**DOI:** 10.3389/fphar.2022.981578

**Published:** 2022-09-15

**Authors:** Fang-Fang He, Yu-Mei Wang, Yi-Yuan Chen, Wei Huang, Zi-Qi Li, Chun Zhang

**Affiliations:** Department of Nephrology, Union Hospital, Tongji Medical College, Huazhong University of Science and Technology, Wuhan, China

**Keywords:** sepsis, autophagy, acute kidney injury, phytochemicals, inflammation

## Abstract

Sepsis is a heterogenous and highly complex clinical syndrome, which is caused by infectious or noninfectious factors. Acute kidney injury (AKI) is one of the most common and severe complication of sepsis, and it is associated with high mortality and poor outcomes. Recent evidence has identified that autophagy participates in the pathophysiology of sepsis-associated AKI. Despite the use of antibiotics, the mortality rate is still at an extremely high level in patients with sepsis. Besides traditional treatments, many natural products, including phytochemicals and their derivatives, are proved to exert protective effects through multiple mechanisms, such as regulation of autophagy, inhibition of inflammation, fibrosis, and apoptosis, etc. Accumulating evidence has also shown that many pharmacological inhibitors might have potential therapeutic effects in sepsis-induced AKI. Hence, understanding the pathophysiology of sepsis-induced AKI may help to develop novel therapeutics to attenuate the complications of sepsis and lower the mortality rate. This review updates the recent progress of underlying pathophysiological mechanisms of sepsis-associated AKI, focuses specifically on autophagy, and summarizes the potential therapeutic effects of phytochemicals and pharmacological inhibitors.

## 1 Introduction

Sepsis is a potentially life-threatening organ dysfunction caused by a dysregulated host response to infection (Singer et al., 2016). Sepsis is characterized by the over-production of pro-inflammatory cytokines such as interleukin (IL)-1β, IL-6, IL-8, and tumor necrosis factor-α (TNF-α), which activate the host’s immune responses. During sepsis, activation of the sympathetic nervous system, endothelial injury, and release of vasoactive substances, such as endothelin, vasopressin, and angiotensin II, cause redistribution of blood flow and disorders of the microcirculation. These factors could damage the kidney tissue and trigger acute kidney injury (AKI) by complex molecular mechanisms. Therefore, sepsis is a principal cause of AKI and accounts for 45%–70% of all AKI events (Uchino et al., 2005). Meanwhile, around 60% of patients with sepsis have AKI (Bagshaw et al., 2009). The characteristics of sepsis-induced AKI include a rapid deterioration of renal function, damaged renal tubular epithelial cells (TECs), and the accumulation of inflammatory cytokines in the kidney, accompanied by multiorgan dysfunction syndromes. Around 15%–20% of patients with AKI will progress to chronic kidney disease, and even develop end-stage renal disease. Understanding the pathophysiology of sepsis-induced AKI facilitates the development of effective therapeutic strategies.

Recently, increasing attention has been paid to the effect of autophagy on septic AKI. Numerous studies have demonstrated that autophagy is activated and plays a renoprotective role in sepsis, although there are some contradictory reports. Autophagy is a normal cell survival phenomenon that maintains intracellular homeostasis by degrading damaged organelles in the lysosome, eliminating pathogens, and sustaining nutritional recycling for metabolic demands (Mizushima and Levine, 2020). Autophagy has been recently highlighted to be a critical mediator for the activation of innate or adaptive immunity to control excessive inflammation (Deretic and Levine, 2018). Inflammation and immune responses have proven to be important in sepsis-induced AKI. Thus, studies on the effect of autophagy might lead to in-depth elucidation of the mechanism of sepsis-associated AKI.

Numerous studies have demonstrated various therapeutic strategies that may be used for treating sepsis including antibiotics, fluid resuscitation, vasopressors, renal replacement therapy, pharmacological inhibitors of signaling pathways, and phytochemicals. Although multiple therapeutic options have been developed for sepsis-induced AKI, the overall mortality is still high. In recent years, many botanical compounds, including phytochemicals and their derivatives, have proven to have multifaceted therapeutic effects on sepsis (Alikiaii et al., 2021). Thus, phytochemicals might be considered as supplementary therapies for sepsis-induced AKI. Many pharmacological inhibitors have also proven to have potential therapeutic effects through different signaling pathways and might enter clinical application in the near future.

In this review, we update recent progress in the underlying pathophysiological mechanisms of sepsis-induced AKI, with specific emphasis on the effect of autophagy, and discuss potential therapies including those targeting autophagy.

## 2 The pathogenesis of sepsis-induced acute kidney injury

Sepsis is a dysregulated host immune response caused by infectious or noninfectious factors that is characterized by over-production of multiple pro-inflammatory cytokines and is associated with multiorgan dysfunction. The infectious factors include bacteria, viruses, fungi, and parasites. The noninfectious conditions include severe acute pancreatitis, serious trauma and so on. According to the degree of severity, it can be classified as sepsis, severe sepsis, and septic shock (Levy et al., 2003). Sepsis refers to the systemic inflammatory response syndrome (SIRS) with infectious or noninfectious etiologies. Severe sepsis indicates sepsis with organ dysfunction. Septic shock is the most serious form characterized by persistent hypotension and organ dysfunction (Poston and Koyner, 2019).

**FIGURE 1:**
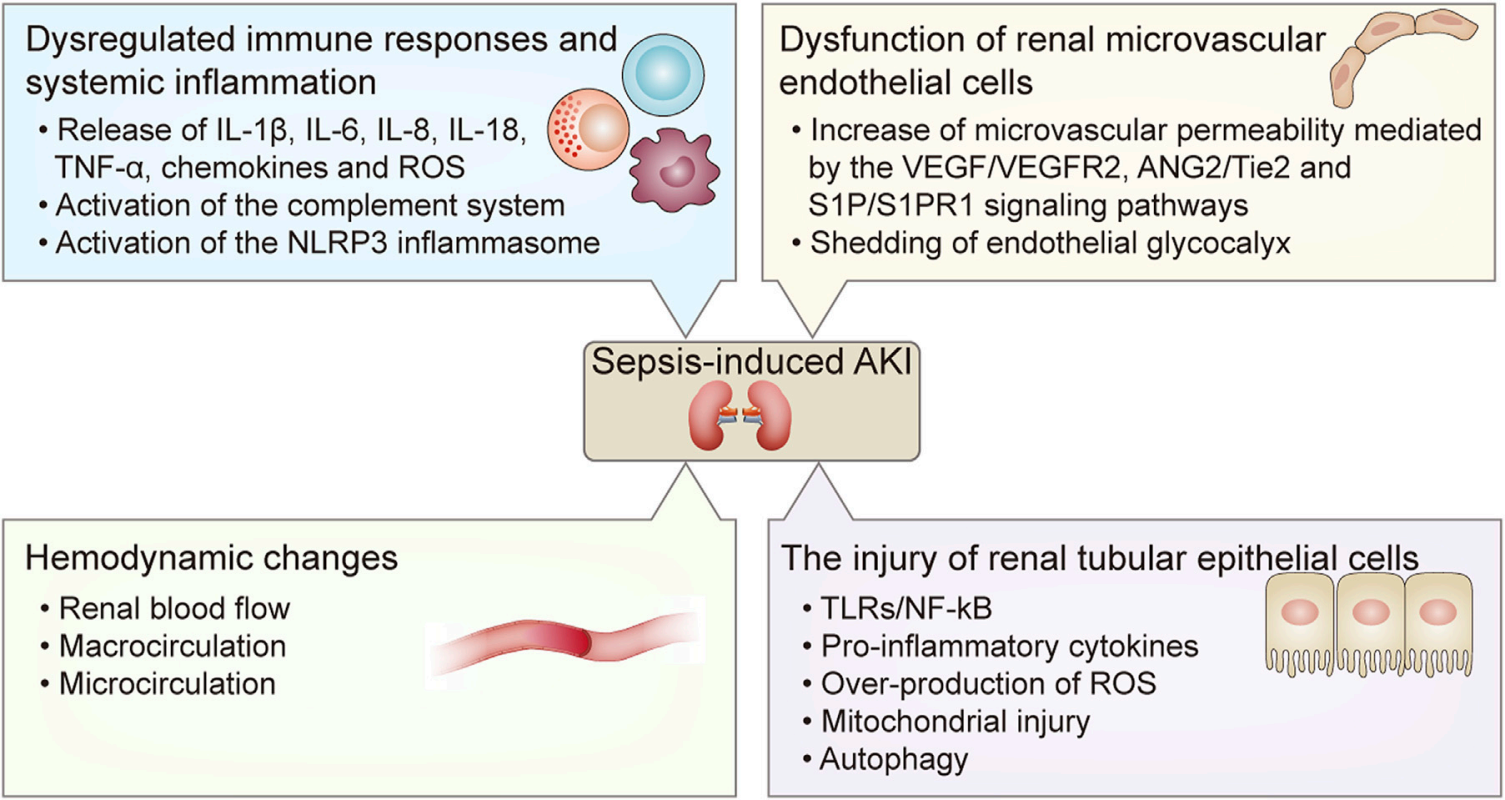
The pathogenesis of sepsis-induced AKI. The pathogenesis of sepsis-induced AKI includes 1) dysregulated immune responses and systemic inflammation including the release of pro-inflammatory cytokines such as IL-1β, IL-6, IL-8, IL-18, TNF-α, chemokines and ROS, and the activation of the complement system and the NLRP3 inflammasome; 2) hemodynamic changes including alterations of renal blood flow, macrocirculation and microcirculation; 3) dysfunction of renal microvascular endothelial cells including increased microvascular permeability mediated by the VEGF/VEGFR2, ANG2/Tie2, and S1P/S1PR1 signaling pathways, and shedding of endothelial glycocalyx; 4) the injury of renal tubular epithelial cells mediated by the TLRs/NF- κB signaling pathway and reduced autophagy at the late stage of sepsis, which result in the release of pro-inflammatory cytokines and over-production of ROS and mitochondrial injury. AKI, acute kidney injury; IL, interleukin; TNF-α, tumor necrosis factor-α; ROS, reactive oxygen species; NLRP3, nucleotide-binding oligomerization domain-like receptor protein 3; VEGF, vascular endothelial growth factor; VEGFR2, VEGF receptor 2; ANG2, Angiopoietin 2; TLRs, Toll-like receptors; NF- κB, nuclear factor kappa B.

AKI is one of the most common and severe comorbidities of sepsis. Septic AKI is a complex and serious clinical syndrome that involves multiple factors, and is associated with high mortality. The clinical features of sepsis-induced AKI are characterized by an abrupt deterioration of renal function that manifests with increased blood urea nitrogen (BUN) and creatinine, and reduced glomerular filtration rate (GFR) and urine output (Bellomo et al., 2017). Nevertheless, the pathogenesis of sepsis-induced AKI has still not been completely elucidated. The current understanding of the pathogenesis of septic AKI includes dysregulated immune response and systemic inflammation, hemodynamic changes, dysfunction of renal microvascular endothelial cells, and injury to the renal TECs (Fani et al., 2018) (Figure 1).

### 2.1 Dysregulated immune response and systemic inflammation

**FIGURE 2:**
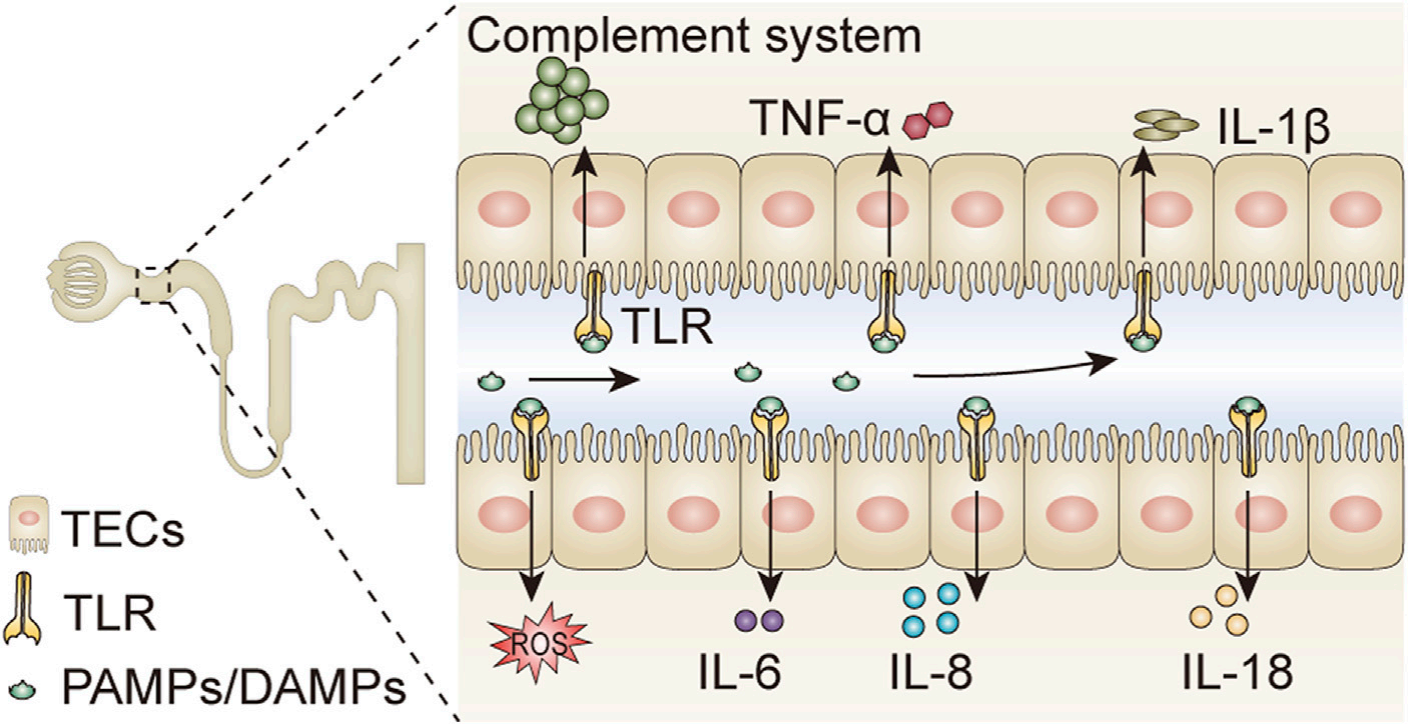
Pathogens activate inflammatory responses in tubular epithelial cells. PAMPs and DAMPs are compounds existing in various pathogens, which can be filtered through the glomerular filtration barrier, and bind to TLRs expressed on tubular epithelial cells. The binding of PAMPs/DAMPs and TLRs promotes the release of pro-inflammatory cytokines including IL-1β, IL-6, IL-8, IL-18, TNF-α, and ROS, and simultaneously activates the complement system. PAMPs, pathogen-associated molecular patterns; DAMPs, damage-associated molecular patterns; IL, interleukin; TNF-α, tumor necrosis factor-α; ROS, reactive oxygen species; TLR, Toll-like receptor; TECs, tubular epithelial cells.

When microbes invade the host, macrophages and innate cellular and humoral immune responses are activated. The innate immune cells recognize pathogens by pathogen-associated molecular patterns (PAMPs) and/or damage-associated molecular patterns (DAMPs) (van der Poll et al., 2017). Lipopolysaccharide (LPS), an element in the cell wall of Gram-negative bacteria, is a member of PAMPs, and is a cardinal cause of sepsis. It is often used for establishing a sepsis-induced AKI model. PAMPs and/or DAMPs are recognized by toll-like receptors (TLRs) that promote the overwhelming release of pro-inflammatory cytokines, including IL-1β, IL-6, IL-8, IL-18, TNF-α, chemokines, and reactive oxygen species (ROS), and simultaneously activate the complement system (Figure 2). The nucleotide-binding oligomerization domain-like receptor protein 3 (NLRP3) inflammasome, a sensor of PAMPs and DAMPs, also plays an important role in inflammatory responses in septic conditions (Li et al., 2022). Dysregulated immune response and systemic inflammation are prominent pathophysiological factors in septic AKI.

### 2.2 Hemodynamic changes

Renal blood flow (RBF) depends on cardiac output and effective circulating volume. During sepsis, cardiac output increases in the early phase, then gradually decreases due to septic injury. A study on ten established septic AKI patients showed the median RBF reduced significantly compared to healthy controls, and this could be detected by cine phase-contrast magnetic resonance imaging (Prowle et al., 2012). Renal hypoperfusion used to be considered a primary cause of AKI. However, this paradigm has been challenged in recent years. A series of studies have found that RBF is preserved or even increased in septic AKI (Langenberg et al., 2006; Prowle et al., 2012; Maiden et al., 2016). Takasu et al. demonstrated that RBF doubled and renovascular resistance decreased at 6 h after cecal ligation and puncture (CLP) compared with the baseline (Leisman et al., 2021). In large animal models, RBF increased in the early stage of sepsis (Di Giantomasso et al., 2003; Langenberg et al., 2007). These findings imply that hypoperfusion of the kidney is not required for the occurrence of septic AKI. The discrepancy in these studies implies that multiple other mechanisms may participate in the pathogenesis of sepsis-induced AKI. The effective circulating volume is decreased owing to systemic vasodilatation and reduced peripheral vascular resistance. The dysfunction of renal vascular endothelial cells and the release of vasoactive substances such as nitric oxide cause dilatation of the blood vessels. These factors lead to macrohemodynamic instability. However, several studies have shown that sepsis occurrs with microcirculatory alterations in the absence of macrocirculatory changes (Post et al., 2017). These findings indicate that microcirculatory hemodynamic instability might contribute to sepsis-induced AKI.

### 2.3 Dysfunction of renal microvascular endothelial cells

The renal vasculature consists of the renal artery, the microvasculature of the glomerulus, and the peritubular capillary, which are distributed in different segments. The homeostasis of the RBF and microvascular permeability is mainly controlled by endothelial cells. A previous study in septic mice observed that endothelial fenestrations were increased, leading to vascular leakage (Escobar et al., 2015). Changes in intercellular contact between the endothelial cells lead to increased microvascular permeability and leakage of leukocytes, which are controlled by intricate molecular interactions. A previous study showed that endothelial nitric oxide synthase was reduced in the CLP-induced septic model, and played a crucial role in vasodilation (Condor et al., 2016). Shedding of the endothelial glycocalyx is observed in all sepsis models, and is paralleled by an increase in soluble glycocalyx components in the plasma. Endothelial damage and the loss of glycocalyx promote leukocyte leakage and platelet adhesion, accompanied by a decrease in blood flow velocity. This might result in the formation of microthrombi and consequent occlusion of the capillaries (Peerapornratana et al., 2019).

Vascular endothelial growth factor (VEGF) and VEGF receptor 2 (VEGFR2) complex play important roles in controlling microvascular permeability. In an LPS-induced septic mouse model, the VEGF levels increased in the plasma for up to 24 h, whereas they decreased in the kidney. These caused a glomerular endothelial injury that manifested as decreased density of the endothelial fenestrae and increased diameter of the remaining fenestrae. This effect is mediated by TNF-α activation of TNF receptor 1 (Xu et al., 2014). However, in the CLP model, though the plasma levels of VEGF increased, they were not accompanied by any changes in the kidney VEGF levels. This discrepancy might be caused by the different animal models. The elevated plasma VEGF levels could activate VEGFR2, which results in the degradation of vascular endothelial (VE)-cadherin, leading to loss of endothelial integrity and an increase in microvascular permeability (Lampugnani et al., 2018). Anti-VEGF therapy might thus prevent microvascular leakage (Jeong et al., 2013).

The angiopoietin (ANG)-Tie2 system is also involved in endothelial dysfunction during sepsis. ANG1 and ANG2 are ligands for the endothelial receptor tyrosine kinase Tie2. ANG1 acts as a Tie2 agonist to maintain vascular stabilization, whereas ANG2 acts as an antagonist. The plasma levels of ANG2 increased, while renal levels of Tie2 and ANG1 decreased after LPS administration (Aslan et al., 2017). The elevated ANG2 caused dephosphorylation of Tie2 and led to vessel destabilization (Parikh, 2017). A previous study demonstrated that pretreatment with an engineered ANG1 construct induced the phosphorylation of Tie2 and alleviated increased renal microvascular permeability (Kim et al., 2009). Binding of sphingosine-1-phosphate (S1P) to S1P receptor 1 (S1PR1) also results in enhanced endothelial permeability (Sanchez, 2016). In septic mice, the plasma levels of S1P were found to be decreased. Pretreatment with an S1PR1 agonist ameliorated the enhanced microvascular permeability (Wang et al., 2015b; Kurano et al., 2018).

Dysfunction of endothelial cells also causes coagulation imbalance and inflammatory cell recruitment through diverse molecular mechanisms (Molema et al., 2022). Therefore, vascular endothelial cells in the kidney are major contributors to the development of sepsis-induced AKI. Understanding the exact role of endothelial cells might help to uncover novel therapeutic strategies.

### 2.4 The injury of renal tubular epithelial cells

Acute TECs injury often occurs in sepsis-induced AKI. TLRs, especially TLR2 and TLR4, are expressed on the TECs. PAMPs and DAMPs are recognized by TLRs located in the apical membrane of TECs, and activate nuclear factor kappa B (NF-κB), resulting in the release of pro-inflammatory cytokines and over-production of ROS, and mitochondrial injury (Hotchkiss and Karl, 2003; Kalakeche et al., 2011). Although these factors aggravate TECs injury, acute tubular necrosis (ATN) was not observed in patients dying of sepsis (Takasu et al., 2013). Thus, sepsis-induced AKI is not equivalent to ATN (Manrique-Caballero et al., 2021). Autophagy is activated in renal proximal TECs in LPS-induced septic mice (Leventhal et al., 2016). A previous study, using a specific proximal tubule marker angiotensin-converting enzyme, found that autophagy was transiently induced in the proximal tubule primarily at 3 h after CLP. However, autophagy was found to be reduced at 9 h until 18 h, and this was accompanied by pathological and functional injury in the kidney (Hsiao et al., 2012). These findings suggest that decreased autophagy in the late stage of sepsis might contribute to the dysfunction of proximal TECs.

## 3 Autophagy in sepsis-induced acute kidney injury

There is increasing evidence for the role of autophagy in sepsis-induced AKI. Autophagy is a conservative cellular homeostatic process that degrades damaged organelles, aggregated proteins, or pathogens, recycles endogenous metabolic precursors such as nucleotides, amino acids, and fatty acids, and reutilizes energy. The process of autophagy includes the formation of autophagosomes, the delivery of cytoplasmic cargoes to the lysosomes, the fusion of autophagosomes and lysosomes to form autophagolysosomes, and the proteolytic degradation of cargoes (Choi et al., 2013). This dynamic process is called “autophagic flux” and is regulated by various autophagy-related genes (Atgs). During autophagy, a cytosolic form of microtubule-associated protein 1 light chain 3 (LC3-I) is conjugated with phosphatidylethanolamine to form lipidated-LC3 (LC3-II). This indicates the formation of an autophagosome and serves as a marker of autophagy. Selective autophagy maintains cellular homeostasis by directed degradation of dysfunctional organelles, including mitochondria (mitophagy), endoplasmic reticulum (ERphagy), peroxisomes (pexophagy), aggregates of proteins (aggrephagy), glycogen (glycophagy), lipids (lipophagy), defective ribosomes (ribophagy), nuclei (nucleophagy), and the removal of microorganisms (xenophagy) (Bhatia and Choi, 2020). Mitophagy is the autophagy of mitochondria, which eliminates damaged mitochondria in TECs and protects renal function during septic AKI (Sun et al., 2019).

Autophagy plays both pro-survival and pro-pathogenic roles in different human diseases. Recent studies suggest that autophagy is a vital modulator of kidney diseases (Choi, 2020). Dysfunction of autophagy implicated in the pathogenesis of sepsis-associated AKI. A previous study showed that LC3-II increased at 3 h, whereas it decreased between 9 and 18 h and this was accompanied by a deterioration in renal function in CLP mice (Hsiao et al., 2012). Another study showed that the expressions of LC3-I/II peaked at 6 and 36 h after CLP (Karagiannidis et al., 2016). These differences might be due to the different ages of the animals chosen and the different time points for measurement. Thus, autophagy is activated in the early stage and exhausted in the late stage of sepsis (Ho et al., 2016).

### 3.1 The effects of autophagy in sepsis-induced acute kidney injury

Autophagy may play protective or injurious roles in different organs during sepsis (Yin et al., 2019). In the kidney, a majority of studies have demonstrated that activation of autophagy plays a renoprotective role in septic AKI (Karagiannidis et al., 2016; Mei et al., 2016; Bhatia and Choi, 2020). Leventhal et al. (2016) showed that proximal tubule-specific Atg7 gene knockout increased the severity of LPS-induced AKI in septic mice. They found that tubular injury was more severe, and that BUN was higher in Atg7 knockout mice than in control mice after LPS injection. Moreover, the expressions of IL-6 and the signal transducer and activator of transcription 3 (STAT3) phosphorylation were increased in Atg7 knockout kidneys as compared with control mice after LPS administration. These results confirm the protective effect of autophagy on sepsis-induced AKI. However, in another study, the authors found that autophagy was activated in septic AKI and resulted in kidney injury. Further, inhibition of autophagy by a pharmacologic autophagy inhibitor 3-methyladenine (3-MA) increased the survival rate in CLP mice (Wu et al., 2019). They concluded that activation of autophagy aggravated kidney damage in septic AKI, whereas inhibition of autophagy had a protective role. The disagreement among these studies might be due to the different effects of autophagy-specific gene conditional knockout models and extensive autophagy inhibition by drugs that may interfere with other signaling pathways.

### 3.2 The regulation of autophagy in sepsis-induced acute kidney injury

#### 3.2.1 Adenosine monophosphate-activated protein kinase/mammalian target of rapamycin signaling pathway

Previous studies have demonstrated that the mammalian target of rapamycin (mTOR) negatively regulates autophagy, and that inhibition of mTOR could initiate autophagy. In a CLP model of sepsis, rapamycin, an mTOR inhibitor, activated autophagy and alleviated sepsis-induced myocardial dysfunction (Hsieh et al., 2011). Adenosine monophosphate-activated protein kinase (AMPK) negatively regulates mTOR; thus, activation of AMPK could suppress mTOR activity, promote autophagy, limit the over-production of pro-inflammatory cytokines, and mitigate sepsis-induced kidney injury (Kim et al., 2011; Escobar et al., 2015). Nevertheless, inhibition of AMPK activation with compound C prevented autophagy and worsened CLP-induced kidney injury (Escobar et al., 2015). These results suggest that the AMPK/mTOR signaling pathway is involved in sepsis-induced kidney injury.

#### 3.2.2 CaMKIV/mammalian target of rapamycin signaling pathway

Calcium/calmodulin-dependent protein kinase (CaMK) has been reported to regulate autophagy in sepsis. Zhang et al. found that the expression of mTOR and the numbers of autophagosomes and autophagolysosomes were reduced in the renal cortex, and cystatin C was increased in LPS-treated CaMKIV knockout mice as compared with control mice. This study concluded that CaMKIV preserved mTOR expression by inhibiting glycogen synthase kinase-3 beta (GSK3β) and F-box and WD repeat-containing protein 7 (FBXW7) mediated ubiquitination and proteasomal degradation, and promoted autophagy in LPS-induced AKI (Zhang et al., 2014). This study countered the current paradigm that inhibition of mTOR augments autophagy. This may be because CaMKIV regulates autophagy, independent of mTOR inhibition. These data suggest that CaMKIV plays a vital role in the regulation of autophagy in LPS-induced AKI.

#### 3.2.3 Sirtuins

Sirtuins (SIRTs) are a family of nicotinamide adenine dinucleotide (NAD^+^)-dependent histone deacetylases composed of seven isoforms (SIRT1-SIRT7), which are involved in many pathophysiological processes including inflammation, fibrosis, atherosclerosis, energy metabolism, aging-related diseases and cancers (Grootaert and Bennett, 2022; Huang et al., 2022; Yang et al., 2022). SIRT1 activity has been shown to be decreased in several septic animal models. Activation of SIRT1 decreased inflammatory cytokines and improved survival of CLP mice (Opal et al., 2016; Xu et al., 2016). SIRT3, located in the mitochondrial matrix, is reported to protect against sepsis-induced AKI through regulating autophagy. Knockout of SIRT3 inhibited autophagy and exacerbated kidney dysfunction in CLP mice. Overexpression of SIRT3 enhanced autophagy, attenuated tubular cell apoptosis and accumulation of pro-inflammatory cytokines in CLP-induced septic AKI. These processes are mediated by the AMPK/mTOR pathway (Zhao et al., 2018). In LPS-induced septic AKI mice, SIRT6 is also involved in autophagy regulation. Overexpression of SIRT6 promoted autophagy, decreased the production of TNF-α and IL-6, and inhibited TECs apoptosis, thus protecting against kidney damage from septic AKI (Zhang et al., 2019). These data suggest that SIRTs participate in the regulation of autophagy in septic AKI and that activation of SIRTs might be a potential therapeutic strategy.

#### 3.2.4 Toll-like receptor 4 signaling pathway

TLR4, a member of pattern recognition receptors that recognize microbial PAMPs, is involved in the regulation of autophagy during sepsis. Previous studies have shown that TLR4 is activated after LPS stimulation and regulates autophagy via the TLR4-myeloid differentiation primary response 88 (MyD88)-mitogen-activated protein kinase (MAPK)/NF-κB and TLR4/Phosphoinositide 3-kinase (PI3K)/v-akt murine thymoma viral oncogene homologue (Akt)/mTOR signaling pathways (Kawai and Akira, 2011; Monlish et al., 2016). A recent study demonstrated that inhibition of TLR4 by resatrovid (TAK242) mitigated sepsis-induced kidney injury through inhibiting autophagy (Li and Feng, 2022). Another study isolated renal TECs from C57Bl/10ScN mice without functional TLR4 and incubated them with LPS. They found that autophagy was not induced in TECs from C57Bl/10ScN mice compared to control mice (Leventhal et al., 2016). This result suggests that TLR4 is required for LPS-induced autophagy in TECs.

#### 3.2.5 Receptor interacting protein kinase 3-transcription factor EB pathway

Receptor interacting protein kinase 3 (RIPK3), a serine/threonine kinase, plays an important role in regulating inflammatory responses (Shlomovitz et al., 2017). A previous study reported that RIPK3 was activated *in vivo* and *in vitro* during septic AKI. Activated RIPK3 inhibited autophagic degradation via suppressing the nuclear translocation and activation of transcription factor EB (TFEB). Inhibition of RIPK3 attenuated tubular damage and improved renal function in sepsis-induced AKI (Li et al., 2021b). These findings demonstrate that the RIPK3-TFEB signaling pathway is involved in autophagic degradation and thus in tubular injury during septic AKI. RIPK3 could therefore be implicated as a negative regulator of autophagy.

## 4 Therapeutic approaches in sepsis-induced acute kidney injury

Severe sepsis often leads to multiple organ dysfunction syndrome (MODS). There are no standardized therapeutic strategies. Most of the current treatments are supportive, and include sustaining hemodynamic stability, managing fluid balance, maintaining acid-base and electrolyte homeostasis, nutritional support including protein and caloric supplements, renal replacement therapy (RRT) with or without hemoperfusion, and plasma perfusion (Ricci et al., 2011). Rivers et al. described early goal-directed therapy that included maintaining central venous pressure ≥8–12 mmHg, mean arterial pressure (MAP) ≥ 65 mmHg, urine output ≥ 0.5 ml/kg/h, and central venous oxygen saturation ≥ 70% (Rivers et al., 2001). Early goal-directed therapy has been shown to improve survival (Rivers et al., 2008). Nevertheless, the mortality of sepsis-induced AKI is still extremely high. Thus, novel therapies are needed urgently to reduce mortality and associated complications. Since the activity of autophagy in proximal TEC is insufficient at the late stage of sepsis, activation of autophagy might be a potential therapy for sepsis-induced AKI. A variety of drugs have been reported to be effective in sepsis-associated AKI by inducing autophagy. Many phytochemicals have anti-inflammatory and anti-oxidant properties, which can be used as a complementary strategy for sepsis-induced AKI. However, these drugs need further comprehensive evaluation.

### 4.1 Antibiotic therapies

Broad-spectrum antibiotics should be administrated within an hour, once sepsis is recognized (Kumar, 2009; Townsend, 2021). Meanwhile, the septic source should be identified so that appropriate antimicrobial therapy may be provided. Some medications, such as vancomycin, aminoglycosides, or amphotericin B, should be used with caution because of their nephrotoxicity. Delayed administration of antibiotics is associated with the early development of AKI (Bagshaw et al., 2009).

### 4.2 Fluid resuscitation and vasopressors

Fluid resuscitation is needed when sepsis-induced tissue hypoperfusion occurs. Balanced crystalloids are considered as the first-line choice for fluid expansion (Patel et al., 2022). Vasopressors should be initiated when fluid resuscitation is insufficient to correct hypotension. A multicenter, open-label clinical trial showed that maintaining an MAP at 65–70 mmHg was optimal for septic patients undergoing resuscitation, while a higher MAP target (80–85 mmHg) did not improve mortality at either 28 or 90 days (Asfar et al., 2014). Norepinephrine (NE) is the first choice to restore blood pressure. On the other hand, vasopressin is not recommended as an alternative option, for it does not reduce the number of kidney failure-free days and has no effect on mortality rates. This was established in a double-blind, randomized clinical trial (RCT) and in a meta-analysis of RCTs (Gordon et al., 2016; Lankadeva et al., 2019; Nagendran et al., 2019). However, restoring the macrocirculation is not sufficient to adequately restore the microcirculation (Holthoff et al., 2012). Several clinical trials found that resuscitation had no effects on the improvement in mortality or RRT requirement (Investigators et al., 2014; Mouncey et al., 2015; Kellum et al., 2016). Thus, restoration of microcirculatory perfusion might be of partial benefit in septic AKI patients. More research is required to determine the role of fluid resuscitation in septic AKI.

### 4.3 Renal replacement therapy

A large clinical trial compared early versus delayed initiation of RRT in critically ill patients with AKI. The results revealed that the one-year all-cause mortality rate was lower, and that the renal recovery rate was higher in the early initiation group as compared with the delayed initiation group (Meersch et al., 2018). Another RCT also showed that early initiation of RRT lowered mortality at 90 days compared with delayed initiation of RRT (Zarbock et al., 2016). However, two other RCTs found that the mortality in the early initiation of the RRT group was similar to that in the delayed initiation of the RRT group (Gaudry et al., 2016; Gaudry et al., 2018). The discrepancies in the results of these clinical trials may be due to different study designs and different clinical conditions. Hemoperfusion with polymyxin B and high-volume hemofiltration has been used for eliminating LPS and inflammatory cytokines, but the vast majority of studies demonstrated that they had no benefit in survival or renal outcome (Zhang et al., 2012; Joannes-Boyau et al., 2013; Payen et al., 2015; Dellinger et al., 2018).

### 4.4 Phytochemicals

Many natural plants contain bioactive chemical monomers that have been reported to have anti-infectious, anti-inflammatory, anti-tumor, neuroprotective, and immunomodulatory properties (Song et al., 2017; Wen et al., 2018; Gao et al., 2020; Wang et al., 2021a; Zhu et al., 2021a; Zhang et al., 2021b; Zhu et al., 2021b; Bai et al., 2022). Here, we will discuss the most studied phytochemicals administrated in sepsis and explore their potential applications in sepsis-induced AKI (Table 1).

**TABLE 1:** Phytochemicals for treating sepsis-induced AKI.

Phytochemicals	Sources	Mechanisms	Effects	References
Resveratrol	Grapes; red wine; berries	Scavenged RNS; Restored SIRT1/3 activity; Reduced acetylated SOD2 levels; Enhanced beclin1 deacetylation-mediated autophagy	Attenuated oxidative stress and mitochondrial injury; Restored renal microcirculation and improved renal function	Kung et al. (2021); Rudrapal et al. (2022); Holthoff et al. (2012); Kitada and Koya. (2013); Xu et al. (2016); Deng et al. (2021)
Ferulic acid	Widely existing in plant cell walls	NF-κB signaling pathway	Suppressed inflammatory cytokines; Increased the antioxidant levels; Attenuated fibrosis; Improved renal function	Mir et al. (2018)
Moringa isothiocyanate-1	Seeds of *Moringa oleifera* Lam	Suppressed nuclear accumulation of NF-κB; Promoted Nrf2 nuclear transport	Mitigated oxidative stress and inflammation	Sailaja et al. (2021)
Curcumin	*Curcuma longa*	Inhibition of lncRNA PVT1; Suppression of the JAK2/STAT3 and JNK/NF-κB signaling pathways; Upregulation of PPARγ.	Decreased serum inflammatory mediators, such as IL-6 and TNF-α; Improved RBF and renal microcirculation	Wang et al. (2021b); Siddiqui et al. (2006); Huang et al. (2020); Zhu et al. (2020); Wang et al. (2015a)
Zingerone	Ginger	Inhibition of the TLR4/NF-κB signaling pathway	Ameliorated tubular dilation and distortion; Attenuated oxidative stress; Inhibited the production of IL-6, TNF-α, IL-1β	Song et al. (2016); Lee et al. (2019)
Rhizoma Coptidis extracts	The root of *Coptis chinensis Makino*	HO-1, NOS2 and PPARα	Inhibited inflammation and oxidative stress	Zheng et al. (2021)
Glycyrrhizic acid	Licorice	ERK/NF-κB signaling pathway	Inhibited the production of TNF-α, IL-1β, and IL-6; Suppressed oxidative stress and apoptosis	Zhao et al. (2016a); Zhao et al. (2016b)
Quercetin	Flavonoids	Activation of SIRT1 and NF-κB; Induction of p53 deacetylation; Promotion of autophagy	Inhibited inflammation and apoptosis; Upregulated antioxidants	Sun et al. (2021); Khajevand-Khazaei et al. (2018); Lu et al. (2021)

RNS, reactive nitrogen species; SIRT, Sirtuin; SOD2, superoxide dismutase 2; NF-κB, nuclear factor kappa B; JAK2, Janus kinase 2; STAT3, signal transducer and activator of transcription 3; JNK, the c-Jun N-terminal kinase; PPARγ, peroxisome proliferator-activated receptor-γ; IL, interleukin; TNF-α, tumor necrosis factor-α; TLR4, Toll-like receptor 4; HO-1, hemeoxygenase-1; NOS2, nitric oxide synthase 2; ERK, extracellular signal regulated kinase.

#### 4.4.1 Resveratrol

Resveratrol is a natural polyphenolic phytoalexin found in grapes, red wine, and berries that has antioxidant and vasodilatory effects in various diseases (Gresele et al., 2011). Numerous studies have shown that resveratrol exerts its antioxidative effects through scavenging ROS and regulating the activity and expression of antioxidant enzymes (Kung et al., 2021; Rudrapal et al., 2022). Holthoff et al. (2012) reported that resveratrol improved the survival of CLP-induced septic mice through restoring renal microcirculation and scavenging reactive nitrogen species. Resveratrol, also considered as a chemical activator of SIRT1, could restore SIRT1/3 activity, reduce acetylated superoxide dismutase 2 levels, attenuate oxidative stress and mitochondrial injury, and finally improve renal function in the septic rat model (Kitada and Koya, 2013; Xu et al., 2016). Activation of SIRT1 by resveratrol protected against sepsis-induced AKI through enhancing beclin1 deacetylation-mediated autophagy (Deng et al., 2021). Therefore, these data suggest that resveratrol could be used as a supplemental treatment for protecting against sepsis-induced AKI.

#### 4.4.2 Ferulic acid

Ferulic acid (FA), a hydroxycinnamic acid that is widely present in plant cell walls, has anti-microbial, antioxidant, and anti-inflammatory properties (Stompor-Goracy and Machaczka, 2021). Mir et al. (2018) demonstrated that FA suppressed inflammatory cytokines, increased antioxidant levels, and thus attenuated fibrosis and improved renal function in mice with LPS-induced AKI mediated by the NF-κB signaling pathway. These results indicate that FA might be considered as a novel treatment for sepsis-induced AKI.

#### 4.4.3 Moringa isothiocyanate-1

Moringa isothiocyanate-1 (MIC-1), derived from the seeds of *Moringa oleifera* Lam, displayed antioxidant and anti-inflammatory activities in several chronic inflammatory disorders (Jaja-Chimedza et al., 2017; Kim et al., 2017). A previous study found MIC-1 inhibited pro-inflammatory cytokines such as IL-6, IL-1β, IFN-α, and TNF-α in the liver, kidney, and spleen of LPS-treated mice. MIC mitigated oxidative stress and inflammation via suppression of nuclear accumulation of NF-κB, and promotion of Nrf2 nuclear transport (Sailaja et al., 2021). These findings indicate that MIC-1 might be a promising phytochemical for ameliorating inflammation in sepsis-related organ dysfunction.

#### 4.4.4 Curcumin

Curcumin, a natural plant phenolic compound isolated from *Curcuma longa*, has been used as a complementary therapeutic agent in hypertension, metabolic syndrome, arthritis, cerebral ischemic, and inflammatory diseases (Menon and Sudheer, 2007; Karimi et al., 2019; Fan and Lei, 2022; Joshi et al., 2022). A recent study demonstrated that curcumin treatment decreased serum inflammatory mediators such as IL-6 and TNF-α, improved RBF and renal microcirculation without any effect on cardiac output, and alleviated renal histopathological injuries compared to the CLP group (Wang et al., 2021b). Other studies demonstrated that curcumin mitigated sepsis-induced AKI through inhibition of long non-coding RNA plasmacytoma variant translocation 1 (lncRNA PVT 1), suppression of the Janus kinase 2 (JAK2)/STAT3 and the c-Jun N-terminal kinase (JNK)/NF-κB signaling pathways, and upregulation of peroxisome proliferator-activated receptor-γ (PPARγ) (Siddiqui et al., 2006; Huang et al., 2020; Zhu et al., 2020). Curcumin-loaded solid lipid nanoparticles are developed due to the poor aqueous solubility and rapid degradation of curcumin. These curcumin-containing nanoparticles showed a better effect on reducing inflammatory factors and alleviating sepsis-induced organ dysfunction (Wang et al., 2015a). These findings suggest that curcumin could be a potential therapeutic agent for treating sepsis-induced AKI.

#### 4.4.5 Zingerone

Zingerone, a phenolic alkenone extracted from ginger, has been proved to exhibit distinct bioactive functions such as anti-inflammatory, anti-cancer, and anti-apoptotic effects (Mahomoodally et al., 2021). Previous studies demonstrated that zingerone alleviated septic AKI by inhibiting the TLR4/NF-κB signaling pathway. Zingerone treatment ameliorated tubular dilatation and distortion, attenuated oxidative stress, and inhibited the production of IL-6, TNF-α, and IL-1β, thus resulting in reduced serum BUN and creatinine and increased survival rate compared to LPS or CLP treated mice (Song et al., 2016; Lee et al., 2019). These results suggest that zingerone could be considered as a therapeutic option for sepsis-induced AKI.

#### 4.4.6 Rhizoma coptidis extracts

Rhizoma coptidis, the root of *Coptis chinensis Makino*, a widely used traditional Chinese herb, has been shown to have anti-bacterial, antioxidant, anti-atherosclerotic, and anti-inflammatory properties in various diseases (Ai et al., 2021; Shao et al., 2021). The active effective components of Rhizoma coptidis extracts include berberine, quercetin, and obacunone. Rhizoma coptidis extracts are proven to inhibit inflammation and oxidative stress, ameliorate renal histological injuries, and improve renal function in sepsis-induced AKI. This process might involve various target proteins and different signaling pathways, including hemeoxygenase-1 (HO-1), nitric oxide synthase 2 (NOS2), and PPARα (Zheng et al., 2021). Thus, Rhizoma coptidis extracts might have a considerable therapeutic effect in sepsis-induced AKI.

#### 4.4.7 Glycyrrhizic acid

Glycyrrhizic acid (GA), a bioactive ingredient of licorice, has been demonstrated to exhibit immunoregulatory, antioxidant, anti-inflammatory, and antiviral properties in various diseases (Li et al., 2021a; Asseri et al., 2022; Qian et al., 2022). Zhao et al. found GA inhibited the production of TNF-α, IL-1β, and IL-6, and suppressed oxidative stress and apoptosis through activation of the extracellular signal-regulated kinase (ERK)/NF-κB signaling pathway in LPS and CLP-induced septic rats (Zhao et al., 2016a; Zhao et al., 2016b). Therefore, GA could decrease the serum BUN and creatinine, improve pathological injuries and increase the survival rate of septic rats. These studies suggest that GA could be used as a new therapeutic approach for sepsis-induced AKI.

#### 4.4.8 Quercetin

Quercetin, a plant flavonoid, has antioxidant, anti-fibrotic, anti-inflammatory, anti-cancer and neuroprotective effects (Ozsoy Gokbilen et al., 2022; Zhang et al., 2022). A previous study found that resveratrol/quercetin induced activation of SIRT1, resulting in the induction of p53 deacetylation. Deacetylated p53 promoted autophagy in renal TECs and attenuated sepsis-induced AKI (Sun et al., 2021). A glycoside of the bioflavonoid quercetin, rutin, has also been proved to protect against endotoxemic renal damage through activation of SIRT1, inhibition of inflammation, and upregulation of antioxidants (Khajevand-Khazaei et al., 2018). Lu et al. (2021) loaded quercetin onto a biodegradable polymer carrier and assessed the function of this nanoparticle in septic AKI mice. The results demonstrated that quercetin pretreatment upregulated the expression of SIRT1, suppressed apoptosis, inflammation, and activation of NF-κB induced by LPS. These data imply that quercetin could be considered as a therapeutic strategy for sepsis-induced AKI.

Many other phytochemicals such as Sargentodaxa cuneata, Cintelactone A, and rosmarinic acid are also proven to have anti-microbial, antioxidant, and anti-inflammatory activities (Zhang et al., 2021a; Zhang et al., 2021c; Di et al., 2021). They have been used for treating various inflammatory-mediated diseases. Although some of them have not been shown to have beneficial effects on the kidney, most of them have anti-inflammatory and immunomodulatory effects in septic conditions. Future research is needed to provide further insights into the potential use of phytochemicals in sepsis-induced AKI patients.

### 4.5 Pharmacological inhibitors of signaling pathways

Recent advances in the development of pharmacological therapies for treating sepsis-induced AKI have focused on the inhibition of relevant signaling pathways. Here, we discuss the potential pharmacological therapeutic strategies for septic AKI (Table 2).

**TABLE 2:** Pharmacological therapies for treating sepsis-induced AKI.

Drugs	Categories	Mechanisms	Effects	References
Angiotensin II	Angiotensin II	AT1R	Reduced the levels of KIM-1; Alleviated oliguria; Prevented the elevation of serum creatinine	Leisman et al. (2021); Khanna et al. (2017); Tumlin et al. (2018)
AP	Bovine-derived AP; Human recombinant AP	De-phosphorylation of LPS and ATP	Increased endogenous creatinine clearance; Reduced RRT requirement and duration	Peters et al. (2015); Heemskerk et al. (2009); Pickkers et al. (2012); Kiffer-Moreira et al. (2014); Peters et al. (2016); Pickkers et al. (2018)
mTOR inhibitors	Rapamycin/sirolimus; temsirolimus	Promoted autophagy	Increased numbers of autophagosomes; Attenuated mitochondrial damage	Sunahara et al. (2018); Howell et al. (2013)
DEX	DEX	Activation of α2-AR; Regulation of p75NTR/p38MAPK/JNK, PI3K/AKT/mTOR, and α2-AR/AMPK/mTOR signaling pathways; Decreased the activation of NLRP3 inflammasome	Reduced renal sympathetic nerve activity; Inhibited vasopressin release; Promoted diuresis and natriuresis; Inhibited oxidative stress and apoptosis; Enhanced autophagy; Downregulated the expressions of IL-1β and 18	Gellai and Edwards. (1988); Miranda et al. (2015); Wang et al. (2020); Zhao et al. (2020); Yang et al. (2020)
RIPK3 inhibitor	GSK’872	Alleviated oxidative stress and mitochondrial dysfunction; Accelerated the degradation of autophagosomes	Induced the formation of autolysosomes; Alleviated tubular injury and renal dysfunction	Sureshbabu et al. (2018); Li et al. (2021b)

AT1R, angiotensin type-1 receptor; KIM-1, kidney injury molecule-1; AP, alkaline phosphatase; LPS, lipopolysaccharide; ATP, adenosine triphosphate; RRT, renal replacement therapy; mTOR, mammalian target of rapamycin; DEX, dexmedetomidine; α2-AR, α2-adrenoreceptor; MAPK, mitogen-activated protein kinase; JNK, the c-Jun N-terminal kinase; PI3K, phosphoinositide 3-kinase; AMPK, adenosine monophosphate-activated protein kinase; NLRP3, nucleotide-binding oligomerization domain-like receptor protein 3; IL, interleukin; RIPK3, Receptor interacting protein kinase 3.

#### 4.5.1 Angiotensin II

Angiotensin II is a vasoconstrictor that acts on angiotensin type-1 receptor (AT1R), which causes constriction of the efferent arteriole more obviously than afferent arteriole, and results in increased GFR (Denton et al., 2000). Leisman et al. (2021) found that the expression of AT1R was decreased in CLP mice. Angiotensin II prevented the elevation of serum creatinine, reduced the levels of kidney injury molecule-1 (KIM-1), and alleviated oliguria in CLP mice. Losartan, a selective AT1R antagonist, exacerbated CLP-induced elevation of BUN and creatinine, and reduced renovascular resistance. A recent clinical trial demonstrated that angiotensin II infusion restored blood pressure in patients with no response to high-dose vasopressors (Khanna et al., 2017). A post hoc analysis of the Angiotensin II for the Treatment of High-Output Shock 3 (ATHOS-3) trial revealed that angiotensin II improved 28-day survival and the MAP response, and reduced the requirement of RRT compared with the placebo group (Tumlin et al., 2018). These conclusions suggest that AT1R-mediated angiotensin II signaling has a renoprotective role in septic AKI and angiotensin II may represent a novel treatment for septic AKI.

#### 4.5.2 Alkaline phosphatase

Alkaline phosphatase (AP) is an endogenous membrane-bound enzyme that has a renal protective effect via de-phosphorylation of LPS and adenosine triphosphate (Peters et al., 2015). In two small phase 2 clinical trials, administration of bovine-derived AP improved renal function as manifested by an increase in endogenous creatinine clearance, and a reduction in RRT requirement and duration in septic AKI patients (Heemskerk et al., 2009; Pickkers et al., 2012). A more stable and biologically active human recombinant AP combining the properties of intestinal and placental AP was thus developed for treating sepsis-induced AKI (Kiffer-Moreira et al., 2014). However, a recent RCT found that the use of human recombinant AP did not result in any improvement in the short-term creatinine clearance rate at 7 days, but renal function was found to have improved on day 21 and 28, and the all-cause 28-day survival was higher (Peters et al., 2016; Pickkers et al., 2018). The conflicting result of this study may be due to the fact that the 7-day timeframe is too short for evaluating renal function changes. These findings imply that administration of AP might be a promising novel therapy for patients with septic AKI. However, further research is needed to assess the long-term effect of human recombinant AP treatment on sepsis-induced AKI.

#### 4.5.3 Mammalian target of rapamycin inhibitors

Pharmacologic mTOR inhibitors, such as rapamycin/sirolimus and temsirolimus are reported to induce the activation of autophagy. Rapamycin prevented CLP-induced renal damage through promotion of autophagy (Sunahara et al., 2018). A previous study demonstrated that temsirolimus could induce autophagy, as evidenced by an increased LC3-II expression, increased numbers of autophagosomes, and less mitochondrial damage observed by electron microscopy, resulting in improved renal function in aged LPS-induced septic AKI mice. Since AMPK negatively regulates mTOR, this study also utilized 5-aminoimidazole-4-carboxamide ribonucleotide (AICAR), an AMPK activator, to promote autophagy, thereby ameliorating renal dysfunction in CLP-induced septic mice (Howell et al., 2013). These studies imply that mTOR inhibitors protect against septic AKI by enhancing autophagy.

#### 4.5.4 Dexmedetomidine

Dexmedetomidine (DEX), a selective α2-adrenoreceptor (α2-AR) agonist, has been widely used in the intensive care unit for its sedative, analgesic, and anxiolytic effects (Lankadeva et al., 2021). Previous studies demonstrated that DEX exerted renoprotective effects through reducing the activation of the renal sympathetic nerve, inhibiting the release of vasopressin, and promoting diuresis and natriuresis (Gellai and Edwards, 1988; Miranda et al., 2015). Recently, DEX was shown to protect against LPS-induced AKI through inhibiting oxidative stress and apoptosis, and enhancing autophagy via the p75 neurotrophin receptor (p75NTR)/p38MAPK/JNK and PI3K/AKT/mTOR signaling pathways (Wang et al., 2020; Zhao et al., 2020). Another study found that DEX decreased the activation of NLRP3 inflammasome, downregulated the expressions of IL-1β and IL-18, ameliorated LPS-induced AKI, and improved renal function by augmenting autophagy. This process was mediated by the α2-AR/AMPK/mTOR signaling pathway (Yang et al., 2020). These studies indicate that besides its traditional effects, DEX has anti-inflammatory, antioxidant, and anti-apoptosis effects and promotes autophagy during sepsis-associated AKI.

#### 4.5.5 Receptor interacting protein kinase 3 inhibitor

RIPK3, a mediator of necroptosis, has been reported to be involved in sepsis-induced AKI. A previous study reported that the expressions of RIPK3 in urine and plasma were increased in patients with sepsis. This study also showed that RIPK3 aggravated kidney injury through promotion of oxidative stress and mitochondrial dysfunction. Deficiency of RIPK3 by genetic deletion alleviated TECs injury (Sureshbabu et al., 2018). Another study found that activation of RIPK3 led to the accumulation of autophagosomes due to insufficient degradation. Pharmacological inhibition of RIPK3 by GSK’872 accelerated the degradation of autophagosomes, induced the formation of autolysosomes, and thus alleviated tubular injury and improved renal function in sepsis-induced AKI (Li et al., 2021b). Therefore, RIPK3 inhibitors might hold potential as a treatment for septic AKI.

## 5 Conclusion

In this review, we discussed the pathogenesis of sepsis-induced AKI and various therapeutic strategies. Multiple cells and organs participate in the pathogenesis of sepsis-induced AKI. Autophagy is an adaptive intracellular response to septic stress, and it might be a novel therapeutic target for sepsis-induced AKI. Additionally, recent studies suggest that many phytochemicals exert anti-microbial, antioxidant, and anti-inflammatory effects in septic AKI, and these might be used for supplementary therapy to improve renal function and survival rate. More clinical trials are needed to assess their safety and efficacy. Further investigations are needed to delineate the underlying signaling mechanisms of sepsis-induced AKI, to find promising potential targets, and to develop novel and effective drugs in order to lower the high mortality rate of sepsis-induced AKI.
