# Supplementary material for: Sepsis-induced AKI: From pathogenesis to therapeutic approaches
Source: Front Pharmacol. 2022 Sep 15;13:981578. doi: 10.3389/fphar.2022.981578 (PMC9522319; doi:10.3389/fphar.2022.981578)
Supplement: Supplementary file 1 [file DataSheet1.docx]

**Table 1 Phytochemicals for treating sepsis-induced AKI**

| **Phytochemicals** | **Sources** | **Mechanisms** | **Effects** | **References** |
| --- | --- | --- | --- | --- |
| Resveratrol | Grapes; red wine; berries | Scavenged RNS;  Restored SIRT1/3 activity;  Reduced acetylated SOD2 levels; Enhanced beclin1 deacetylation-mediated autophagy | Attenuated oxidative stress and mitochondrial injury;  Restored renal microcirculation and improved renal function | Kung et al., 2021; Rudrapal et al., 2022; Holthoff et al., 2012; Kitada and Koya, 2013; Xu et al., 2016;  Deng et al., 2021 |
| Ferulic acid | Widely existing in plant cell walls | NF-κB signaling pathway | Suppressed inflammatory cytokines; Increased the antioxidant levels; Attenuated fibrosis; Improved renal function | Mir et al., 2018 |
| Moringa isothiocyanate-1 | Seeds of *Moringa oleifera* Lam | Suppressed nuclear accumulation of NF-κB; Promoted Nrf2 nuclear transport | Mitigated oxidative stress and inflammation | Sailaja et al., 2021 |
| Curcumin | *Curcuma longa* | Inhibition of lncRNA PVT1; Suppression of the JAK2/STAT3 and JNK/NF-κB signaling pathways; Upregulation of PPARγ. | Decreased serum inflammatory mediators, such as IL-6 and TNF-α;  Improved RBF and renal microcirculation | Wang et al., 2021b; Siddiqui et al., 2006; Huang et al., 2020;  Zhu et al., 2020;  Wang et al., 2015a |
| Zingerone | Ginger | Inhibition of the TLR4/NF-κB signaling pathway | Ameliorated tubular dilation and distortion; Attenuated oxidative stress; Inhibited the production of IL-6, TNF-α, IL-1β | Song et al., 2016;  Lee et al., 2019 |
| Rhizoma Coptidis extracts | The root of *Coptis chinensis Makino* | HO-1, NOS2 and PPARα | Inhibited inflammation and oxidative stress | Zheng et al., 2021 |
| Glycyrrhizic acid | Licorice | ERK/NF-κB signaling pathway | Inhibited the production of TNF-α, IL-1β, and IL-6; Suppressed oxidative stress and apoptosis | Zhao et al., 2016a;  Zhao et al., 2016b |
| Quercetin | Flavonoids | Activation of SIRT1 and NF-κB;  Induction of p53 deacetylation; Promotion of autophagy | Inhibited inflammation and apoptosis; Upregulated antioxidants | Sun et al., 2021; Khajevand-Khazaei et al., 2018;  Lu et al., 2021 |

RNS: reactive nitrogen species; SIRT: Sirtuin; SOD2: superoxide dismutase 2; NF-κB: nuclear factor kappa B; JAK2: Janus kinase 2; STAT3: signal transducer and activator of transcription 3; JNK: the c-Jun N-terminal kinase; PPARγ: peroxisome proliferator-activated receptor-γ; IL: interleukin; TNF-α: tumor necrosis factor-α; TLR4: Toll-like receptor 4; HO-1: hemeoxygenase-1; NOS2: nitric oxide synthase 2; ERK: extracellular signal regulated kinase.

**Table 2 Pharmacological therapies for treating sepsis-induced AKI**

| **Drugs** | **Categories** | **Mechanisms** | **Effects** | **References** |
| --- | --- | --- | --- | --- |
| Angiotensin II | Angiotensin II | AT1R | Reduced the levels of KIM-1; Alleviated oliguria;  Prevented the elevation of serum creatinine | Leisman et al., 2021; Khanna et al., 2017; Tumlin et al., 2018 |
| AP | Bovine-derived AP;  Human recombinant AP | De-phosphorylation of LPS and ATP | Increased endogenous creatinine clearance;  Reduced RRT requirement and duration | Peters et al., 2015; Heemskerk et al., 2009; Pickkers et al., 2012; Kiffer-Moreira et al., 2014; Peters et al., 2016; Pickkers et al., 2018 |
| mTOR inhibitors | Rapamycin/sirolimus; temsirolimus | Promoted autophagy | Increased numbers of autophagosomes; Attenuated mitochondrial damage | Sunahara et al., 2018; Howell et al., 2013 |
| DEX | DEX | Activation of α2-AR;  Regulation of p75NTR/p38MAPK/JNK, PI3K/AKT/mTOR, and α2-AR /AMPK/ mTOR signaling pathways; Decreased the activation of NLRP3 inflammasome | Reduced renal sympathetic nerve activity;  Inhibited vasopressin release;  Promoted diuresis and natriuresis;  Inhibited oxidative stress and apoptosis; Enhanced autophagy;  Downregulated the expressions of IL-1β and 18 | Gellai and Edwards, 1998;  Miranda et al., 2015;  Wang et al., 2020;  Zhao et al., 2020;  Yang et al., 2020 |
| RIPK3 inhibitor | GSK’872 | Alleviated oxidative stress and mitochondrial dysfunction; Accelerated the degradation of autophagosomes | Induced the formation of autolysosomes; Alleviated tubular injury and renal dysfunction | Sureshbabuet al., 2018;  Li et al., 2021b |

AT1R: angiotensin type-1 receptor; KIM-1: kidney injury molecule-1; AP: alkaline phosphatase; LPS: lipopolysaccharide; ATP: adenosine triphosphate; RRT: renal replacement therapy; mTOR: mammalian target of rapamycin; DEX: dexmedetomidine; α2-AR: α2-adrenoreceptor; MAPK: mitogen-activated protein kinase; JNK: the c-Jun N-terminal kinase; PI3K: phosphoinositide 3-kinase; AMPK: adenosine monophosphate-activated protein kinase; NLRP3: nucleotide-binding oligomerization domain-like receptor protein 3; IL: interleukin; RIPK3: Receptor interacting protein kinase 3.
